# Disentangling transport movement patterns of trucks either transporting pigs or while empty within a swine production system before and during the COVID-19 epidemic

**DOI:** 10.3389/fvets.2023.1201644

**Published:** 2023-07-14

**Authors:** Catalina Picasso-Risso, Carles Vilalta, Juan Manuel Sanhueza, Mariana Kikuti, Mark Schwartz, Cesar A. Corzo

**Affiliations:** ^1^Department of Veterinary Population Medicine, University of Minnesota, Saint Paul, MN, United States; ^2^Facultad de Veterinaria, Universidad de la Republica, Montevideo, Uruguay; ^3^Department of Veterinary Preventive Medicine, College of Veterinary Medicine, The Ohio State University, Columbus, OH, United States; ^4^Unitat mixta d'Investigació IRTA-UAB en Sanitat Animal, Centre de Recerca en Sanitat Animal, Campus de la Universitat Autònoma de Barcelona, Bellaterra, Spain; ^5^IRTA, Programa de Sanitat Animal, Centre de Recerca en Sanitat Animal, Campus de la Universitat Autònoma de Barcelona, Bellaterra, Spain; ^6^Departamento de Ciencias Veterinarias y Salud Publica, Facultad de Recursos Naturales, Universidad Católica de Temuco, Temuco, Chile

**Keywords:** transport, pork industry, network analysis, disease surveillance, Midwest, USA

## Abstract

Transport of pigs between sites occurs frequently as part of genetic improvement and age segregation. However, a lack of transport biosecurity could have catastrophic implications if not managed properly as disease spread would be imminent. However, there is a lack of a comprehensive study of vehicle movement trends within swine systems in the Midwest. In this study, we aimed to describe and characterize vehicle movement patterns within one large Midwest swine system representative of modern pig production to understand movement trends and proxies for biosecurity compliance and identify potential risky behaviors that may result in a higher risk for infectious disease spread. Geolocation tracking devices recorded vehicle movements of a subset of trucks and trailers from a production system every 5 min and every time tracks entered a landmark between January 2019 and December 2020, before and during the COVID-19 pandemic. We described 6,213 transport records from 12 vehicles controlled by the company. In total, 114 predefined landmarks were included during the study period, representing 5 categories of farms and truck wash facilities. The results showed that trucks completed the majority (76.4%, 2,111/2,762) of the recorded movements. The seasonal distribution of incoming movements was similar across years (*P* > 0.05), while the 2019 winter and summer seasons showed higher incoming movements to sow farms than any other season, year, or production type (*P* < 0.05). More than half of the in-movements recorded occurred within the triad of sow farms, wean-to-market stage, and truck wash facilities. Overall, time spent at each landmark was 9.08% higher in 2020 than in 2019, without seasonal highlights, but with a notably higher time spent at truck wash facilities than any other type of landmark. Network analyses showed high connectivity among farms with identifiable clusters in the network. Furthermore, we observed a decrease in connectivity in 2020 compared with 2019, as indicated by the majority of network parameter values. Further network analysis will be needed to understand its impact on disease spread and control. However, the description and quantification of movement trends reported in this study provide findings that might be the basis for targeting infectious disease surveillance and control.

## Introduction

Ground transportation of pigs between sites within a production system occurs frequently for age category and production phase segregation because it has been shown to increase production efficiency and animal health. Although trucks and trailers are designed to load batches of animals with protocols that safeguard animals' wellbeing, health, and safety, it is well-established that animal movements are a source of transmission of infectious disease within the swine industry (1–6), and vehicles which transported animals need to be correctly cleaned and disinfected before loading the next batch of pigs to prevent the risk of pathogen spread (7, 8).

With the intent to mitigate the risk of potential disease spread among pig farms, biosecurity practices such as isolation of incoming breeding stock, testing before commingling animals, shower-in/shower-out, disinfection, and drying rooms for supply entry are recommended among others (9). In addition, cleaning and disinfection of trucks and trailers transporting pigs between loads is an area of focus especially when vehicles transport gilts, boars, recently weaned pigs, and culled animals. Although all these biosecurity measures are useful, they are not completely effective as these depend on a combination of standardized processes and their consistent implementation (9). As an example, it has been described that washing and disinfecting vehicles without the appropriate drying has a similar risk for disease transmission as unwashed vehicles (7, 8). Furthermore, a recent study indicated that PRRSv transmission associated with movements of trucks used for feed and personnel transportation is not negligible (4), adding a layer of complexity when understanding disease spread and designing risk-mitigation (e.g., biosecurity) practices for the swine industry.

Social network analysis has proven to be a useful tool for understanding infectious disease dissemination among populations by analyzing pig movement patterns among animal production systems (10–14). Disease surveillance and control have been traditionally assessed by tracing animal movements as a source of disease spread since infected animals are the most frequent way to spread pathogens to susceptible populations. Furthermore, the potential impact of pathogen dissemination through animals in the US has been assessed *in silico* using retrospective shipment records (4) that can introduce bias in the analysis, when dealing with missing or incomplete data. Limited studies have focused on understanding the potential roles that the vehicles used for feed transport and the personnel involved may have on pathogen dissemination (4, 15, 16), but in fact, the majority of studies have overlooked the role that trucks may have on pathogen spread or assumed zero risk for disease transmission when vehicles visit a site after stopping in a cleaning station (i.e., truck wash station) (4, 6).

Here, as a first step to understanding the role of trucks transporting pigs or empty-unwashed trucks in the spread of diseases, we described and analyzed for the first time the vehicle movement patterns and network structure of one of the largest multi-site pig production systems in the Midwestern US using GPS trackers and including vehicles in the network, regardless of the load status or origin/destination of the movement. We assessed the utility of GPS trackers as a reliable source to record truck movements, characterized vehicle network before and during the COVID-19 pandemic, fidelity (defined as the consistency of vehicle movements over time), and time spent at each site, with the ultimate goal of understanding movement trends that will help to identify potential risky behaviors and spatio-temporal variabilities that can inform epidemic-preparedness and support decisions to improve biosecurity practices and compliance for reducing the spread of diseases compromising swine health.

## Materials and methods

### Population and data source

**Figure 1:**
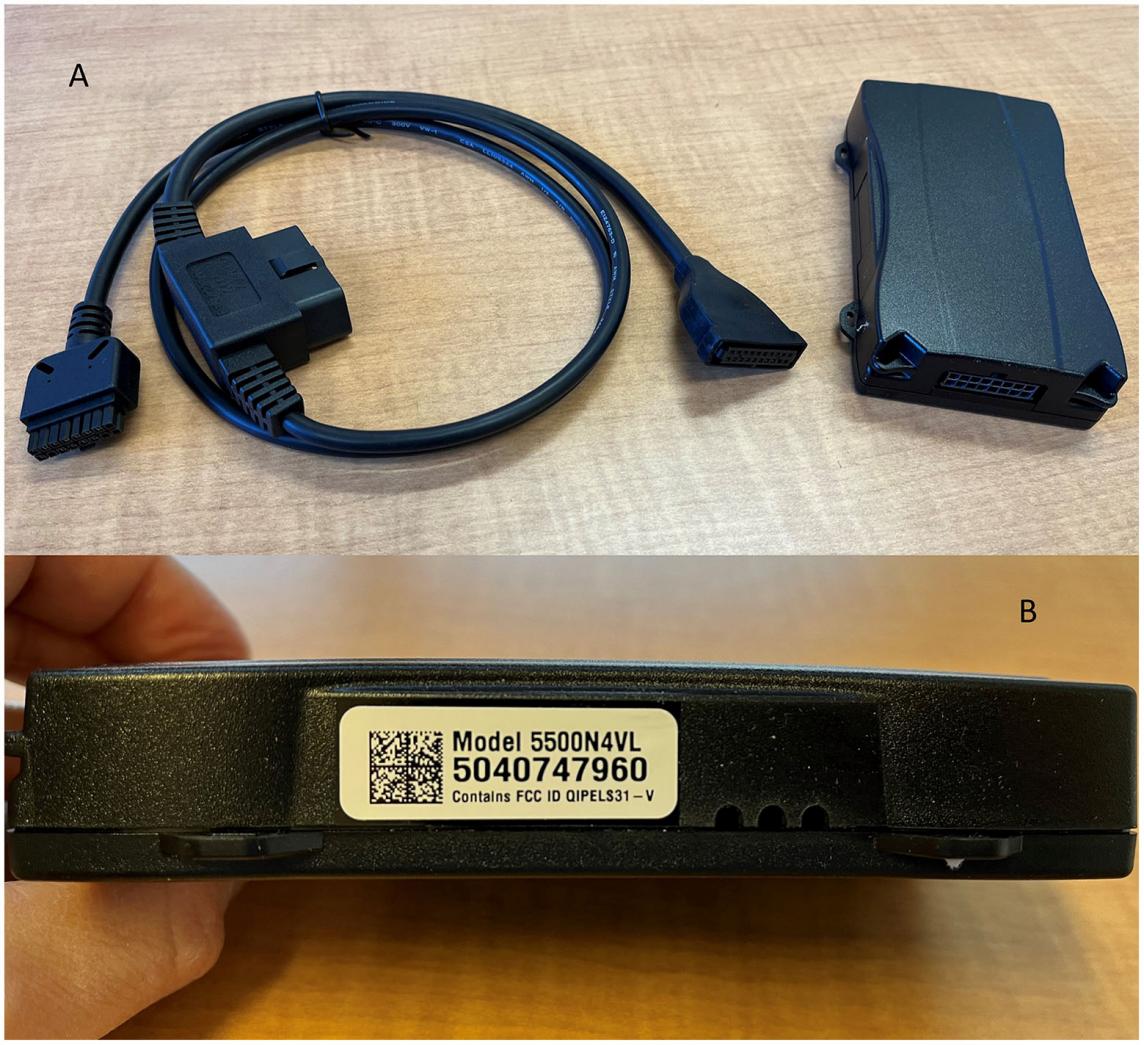
Geolocation tracking devices installed in the vehicles **(A, B)**. Full description of the devices is available online at: https://www.verizon.com/about/news/verizon-expands-asset-tracking-portfolio-introduction-networkfleet-asset-guard-0.

Data were collected from a pig-producing company participating in the University of Minnesota Morrison Swine Health Monitoring Project (MSHMP) (17) that voluntarily agreed to participate in the study. The selected company is a typical multi-site system representative of modern pig production systems in the US This company accounts for approximately 1% of the swine production in the country. The system agreed to have a selected subset of 12 out of 128 owned pig transportation vehicles monitored through geolocation-tracking devices installed on seven trucks transporting only recently weaned pigs and five trailers that transport either gilts or culled sows. On the trailers, the Verizon Networkfleet Asset Guard was attached through screws, whereas the Verizon Networkfleet 5000 Series Model 5500N4VL was connected to the onboard diagnostics (OBD) port on the tractor cab. These devices (Figure 1) are designed to endure long periods of outdoor or other rugged environments while maintaining communication and GPS signal during the journey and through vehicle washing.

Vehicle movement monitoring occurred between January 2019 and December 2020. A total of 6,213 records of vehicles entering 114 georeferenced pig sites were initially considered for this study over the study period (3,659 and 2,554 in 2019 and 2020, respectively). Connections among sites (i.e., vehicle movements) were recreated based on the time and date of entry to each of the geofences and the trajectory of the vehicles tracked.

### Spatiotemporal characterization of the movements

We described the frequency of incoming movement patterns yearly, seasonally, and weekly over the study period by source farm (e.g., farm production type) and time spent by the vehicles at each site. Farm production type was categorized as either sow, nursery, finisher, wean-to-market, or gilt development unit. Differences in the frequency of movements were assessed with univariable and multivariable negative binomial regression which allowed adjusting for over-dispersed data (e.g., frequency of incoming movements mean = 18.71, variance = 274.47) (18) and adjusted for multiple comparisons. We described the proportion of movement performed between pairs of sites grouped by production category (e.g., sow to nursery, sow to sow) and vehicle fidelity over time. Incoming movement density was mapped by year to identify areas of major movement activities.

### Network nomenclature and metrics definition

A yearly static directed non-weighted network was constructed for the complete period (2019–2020). Network nodes or vertices (i.e., elements or units of the network) (19) were polygons created within the extension of preexisting system site locations (i.e., landmarks), which included the abovementioned five different categories of farms and truck wash facilities. The movement of vehicles was captured when tracking devices located in the vehicles that entered or exited each predefined polygon, and thus, the time spent within the polygon was recorded for each vehicle and node.

Edges were created by linking nodes sequentially visited by each vehicle. We selected those nodes in which a stop was recorded for more than 5 min as these were considered an “operational stop”, such as loading/unloading animals or washing the vehicle. When vehicles showed a recorded stop in a node for < 5 min, this was considered a drive-by or an incorrect record (i.e., a result of rearranging vehicles for loading/unloading animals or inaccurately captured by the geofences surrounding the nodes-polygons when the vehicle was circulating close to the polygon boundaries). Furthermore, movements recorded by vehicles entering sequentially onto the same node-polygon for a period of < 15 min within an hour and without visiting any other node in between were merged and analyzed as one movement (i.e., loop to the same node). Additionally, those vehicles stopping for more than 24 h at the nodes were considered as “parked” and not performing an “operational activity” as they could be under maintenance or quarantine (e.g., downtime). The latter situation was included in the network analysis but excluded from our temporal analysis since they did not represent daily routine operation and the time spent at each node can be overestimated.

Network characteristics and features were described by the calculation of (a) network parameters, including the number of nodes and edges, average path length, diameter, edge density, and clustering coefficient, and (b) centrality metrics, such as degree (in and out), closeness (in and out), and betweenness for each node in the networks created. Definitions for each network parameter and metrics have been reviewed and described in a previous study by Martínez-López et al. (19). In brief, *average path length* is the average number of additional nodes contacted or visited in the trajectory to go from node_i_ to node_j_ in the network, *diameter* is the length of the shortest path within the two most distant nodes in the network (i.e., nodes that need the largest number of intermediate nodes to be able to connect each other), *edge density* indicates the proportion of contacts that occur in the network over the potential ones happening, and *clustering coefficient* (i.e., transitivity) is the frequency of loops connecting nodes reciprocally between each other (5). The *degree* is the number of contacts that each node has overall and, when directionality is applied, the number of connections originated (*out-degree*) or received (*in-degree*) by that node. *Closeness* is an estimate of how closely one node is connected to every other node in the network based on all incoming (*in-closeness*) or outgoing (*out-closeness*) connections, and *betweenness* represents the number of times certain node lies on the shortest paths when all the shortest paths are traced between nodes in the network.

To identify cohesive groups in the network, we recognized fully connected nodes in the network (i.e., *cliques*) and densely connected nodes (i.e., *communities*) across the networks constructed. Communities were detected using the algorithm base on propagating labels as an efficient and ease-of-implementation algorithm (20). In brief, unique labels are assigned initially to each node, and after each iteration of the algorithm, new labels are adopted by the nodes, based on the most frequent labels of the neighbors until they converge into one label which represents the community. In the case of ties among neighboring labels, one is randomly selected before the next algorithm iteration.

All statistical analyses were performed using R Studio v4.1.0 software (21). Network creation, description, parameter calculations, and visualization were performed via the igraph package v1.2.6 (22).

## Results

### Site and movement descriptive results

From the predefined 114 sites (i.e., nodes), 12 vehicles moved among them within 2 years of the study period, comprising 12 sow farms, 8 gilt development units (GDU), 17 nurseries, 71 wean-to-market (WTM), 4 finishers (i.e., sites/unique nodes), and 2 truck wash facilities. The nodes were located in Minnesota, South Dakota, Nebraska, and Iowa. After excluding 26% of the movements recorded as described earlier, 4,579 movements were analyzed, with 60% (2,749/4,579) of them occurring in 2019 and the remaining 40% (1,830/4,579) in 2020.

### Spatiotemporal results

The monthly and seasonal in-movement counts were similar across 2019 and 2020 (negative binomial regression *P* > 0.05), with 10–15% of the sites performing at least one movement weekly. The lowest frequency of movements was observed in weeks 70–78 of the study (i.e., May and June 2020, Figure 2). Movements toward sow farms in the winter and summer of 2019 were significantly higher (*P* < 0.05) than movements toward any other farm category or season-year with mean risk ratios ranging from 1.10 to 2257.34. Furthermore, there were fewer movements to finishers in spring 2020 (*P* < 0.01) than any other category or season-year, with risk ratios ranging from 0.0018 to 0.5 (Supplementary Table 1). The seven trucks performed the majority of the movements in both years studied, with 76.8% (2,111/2,749) in 2019 and 66.99% (1,226/1,830) in 2020, while the five trailers did the rest of the movements, 638 in 2019 and 604 in 2020.

**Figure 2:**
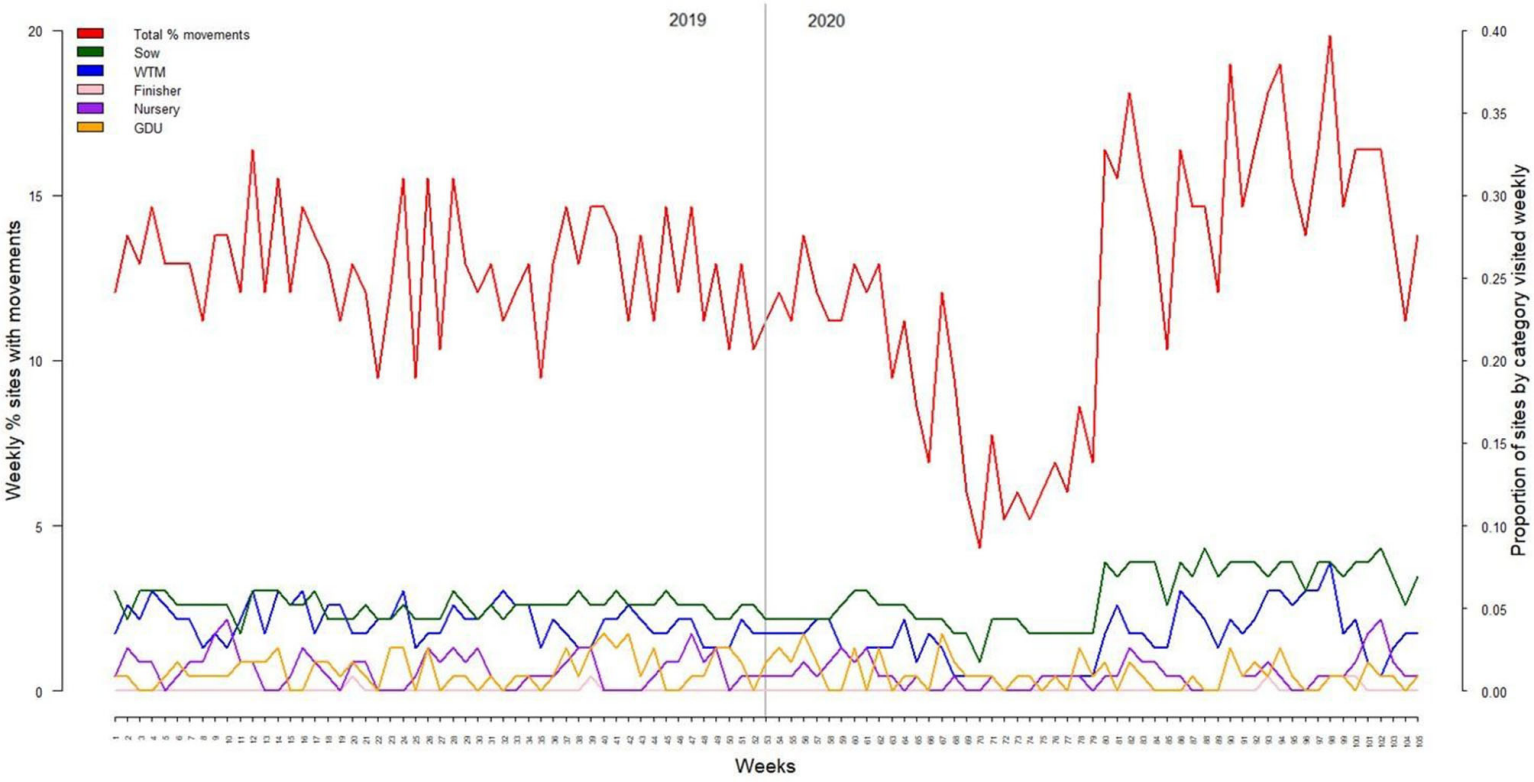
Weekly in-movement distribution by farm categories (proportion) and in total (%) for 2019 **(left)** and 2020 **(right)**.

**Figure 3:**
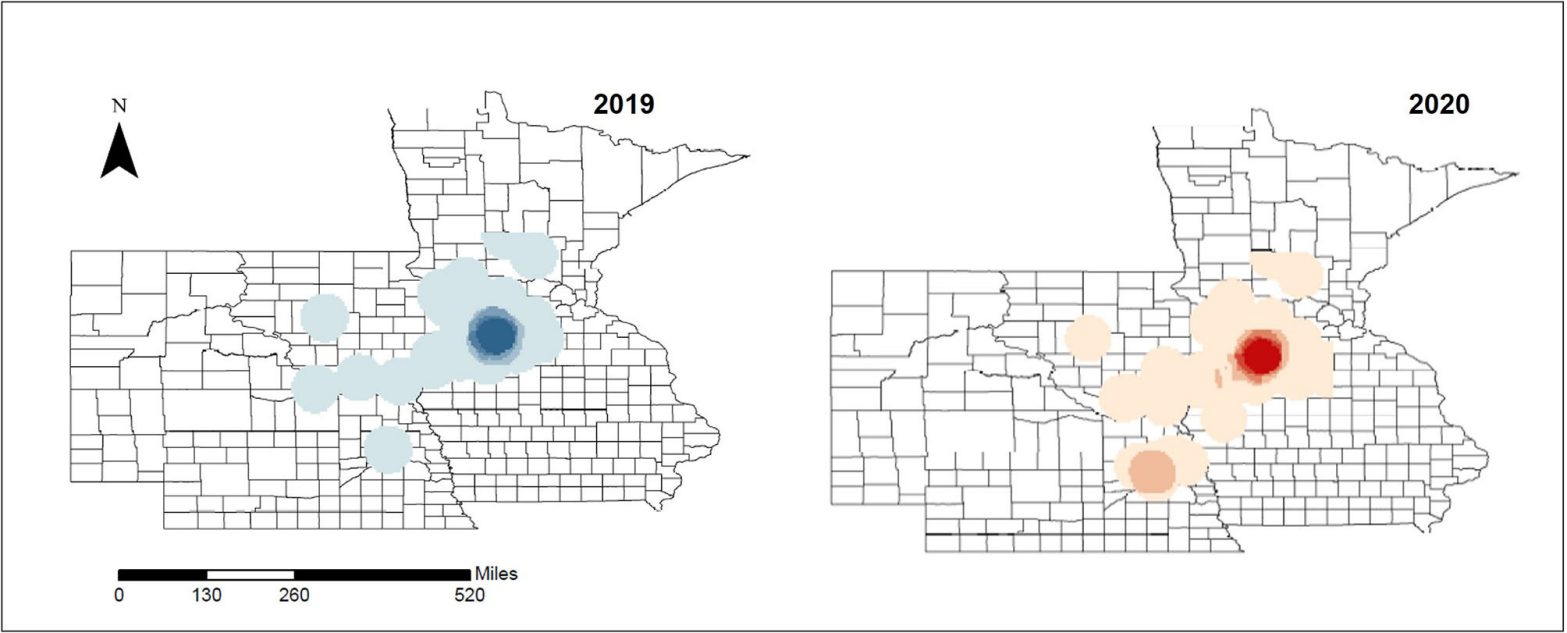
Density of incoming movements within the study area. Higher (dark) and lower (light) density areas (movements per county) are represented for 2019 and 2020, respectively.

In-movements classified by production type dyad indicated that 55.43% of the in-movements were performed by three dyads, sow farm to WTM sites (19.76%/4,579), WTM to truck wash (14.48%), and truck wash to sow farm (20.92%). The remaining in-movements were performed by multiple dyads (Table 1 and Supplementary Table 2). The spatial distribution of movements was consistently higher in the central area of the Midwest states analyzed for both years mapped (Figure 3).

**Table 1:** Frequency of in-movements by farm-type (%). Graded scale colors represent a higher (red) and lower (white) percentage of movements across dyad connections in the 2 years of study (2019–2020).

**Origin/ destination**	**GDU**	**NURSERY**	**SOW**	**TRUCK WASH**	**WTM**	**FINISHER**
FINISHER	0.07	0.02	0.04	0.07	0.00	0.00
GDU	1.62	0.07	1.81	0.07	0.02	0.00
NURSERY	0.20	1.62	1.18	3.49	0.28	0.00
SOW	1.27	4.02	5.48	5.87	19.76	0.00
TRUCK WASH	0.50	0.63	20.92	2.42	2.07	0.00
WTM	0.02	0.44	7.29	14.48	1.05	0.00

GDU, Gilt development unit, WTM, Wean to Market.

Overall, time spent in the nodes was higher (RR: 1.1, *P* < 0.03) in 2020 than in 2019, with higher time spent at truck wash sites in 2020 but without any significant variation observed when analyzed by season (Table 2). However, time spent at truck wash facilities was significantly higher (*P* < 0.001) than any other site categories, with the occurrence of this category being 3.32, 5.83, 14.86, 16.00, and 21.19 times higher, on average, than sow farms, GDU, finishers, nursery, and WTM sites, respectively.

**Table 2:** Median (max-min) minutes spent in each node (i.e., sites) overall by production type and by season during the study period.

**Production type**	**FINISHER**		**GDU**		**NURSERY**		**SOW**		**TRUCK WASH**		**WTM**		**Overall**	
**Stop time**	**Median (Min, Max)**	***n***	**Median (Min, Max)**	***n***	**Median (Min, Max)**	***n***	**Median (Min, Max)**	***N***	**Median (Min, Max)**	***n***	**Median (Min, Max)**	***n***	**Median (Min, Max)**	***n***
Winter19^a^			44.0 (6.00, 602)	25	36.0 (14.0, 76.0)	66	28.0 (6.00, 1,220)	215	162 (11.0, 1,360)	173	26.0 (9.00, 75.0)	150	36.0 (6.00, 1,360)	629
Spring19	50.0 (50.0, 50.0)	1	64.0 (6.00, 478)	25	36.0 (9.00, 60.0)	32	26.0 (8.00, 1,290)	246	149 (9.00, 1,410)	195	22.0 (6.00, 146)	167	32.0 (6.00, 1,410)	666
Summer19	NA		60.0 (18.0, 160)	15	29.0 (10.0, 46.0)	62	26.0 (6.00, 1,280)	267	149 (8.00, 1,440)	210	20.0 (6.00, 101)	171	32.0 (6.00, 1,440)	725
Fall19	20.0 (20.0, 20.0)	1	44.0 (10.0, 148)	34	30.0 (8.00, 76.0)	43	27.5 (6.00, 1,260)	244	152 (6.00, 1,390)	187	20.0 (10.0, 64.0)	167	32.0 (6.00, 1,390)	676
2019	35.0 (20.0, 50.0)	2	54.0 (6.00, 602)	99	32.0 (8.00, 76.0)	210	27.0 (6.00, 1,290)	992	150^+^ (6.00, 1,440)	779	22.0 (6.00, 146)	668	34.0 (6.00, 1,440)	2,750
Winter20^b^			70.0 (8.00, 167)	25	26.0 (6.00, 60.0)	39	28.0 (6.00, 1,270)	173	870 (11.0, 1,420)	137	24.0 (10.0, 179)	116	34.0 (6.00, 1,420)	490
Spring20	NA		98.0 (26.0, 229)	13	22.0 (16.0, 128)	12	31.0 (6.00, 1,280)	112	127 (10.0, 1,350)	73	20.0 (10.0, 66.0)	43	42.0 (6.00, 1,350)	253
Summer20	NA		110 (38.0, 155)	15	31.0 (14.0, 88.0)	24	43.5 (6.00, 1,240)	218	733 (6.00, 1,430)	125	24.5 (6.00, 94.0)	120	45.0 (6.00, 1,430)	502
Fall20	32.0 (9.00, 58.0)	7	77.0 (44.0, 167)	16	31.0 (6.00, 77.0)	40	51.0 (7.00, 1,330)	233	459 (6.00, 1,440)	136	26.0 (8.00, 80.0)	144	47.0 (6.00, 1,440)	576
Winter21^c^			110 (110, 110)	1	25.0 (24.0, 26.0)	2	44.5 (18.0, 1,260)	30	185 (12.0, 1,350)	13	28.0 (14.0, 59.0)	17	44.0 (12.0, 1,350)	63
2020	32.0 (9.00, 58.0)	7	81.0 (8.00, 229)	70	26.0 (6.00, 128)	110	40.0 (6.00, 1,330)	746	345^+^ (6.00, 1,440)	470	24.0 (6.00, 179)	427	42.0 (6.00, 1,440)	1,830
Overall	32.0 (9.00, 58.0)	9	60.0 (6.00, 602)	169	30.0 (6.00, 128)	320	32.0 (6.00, 1,330)	1,738	176^*^ (6.00, 1,440)	1,249	22.0 (6.00, 179)	1,095	36.0 (6.00, 1,440)	4,580

*n*, number of stops per season and production type. Multivariate negative binomial is significantly different (*P* < 0.05), (^*^) from other production types overall or between estimates from 2019 and 2020 (+). GDU, Gilt development unit, WTM, Wean to Market. a, Winter 19 includes January and February 2019; b, Winter 20 includes December 2019, January and February 2020; c, Winter 21 includes December 2020.

### Network characterization

**Figure 4:**
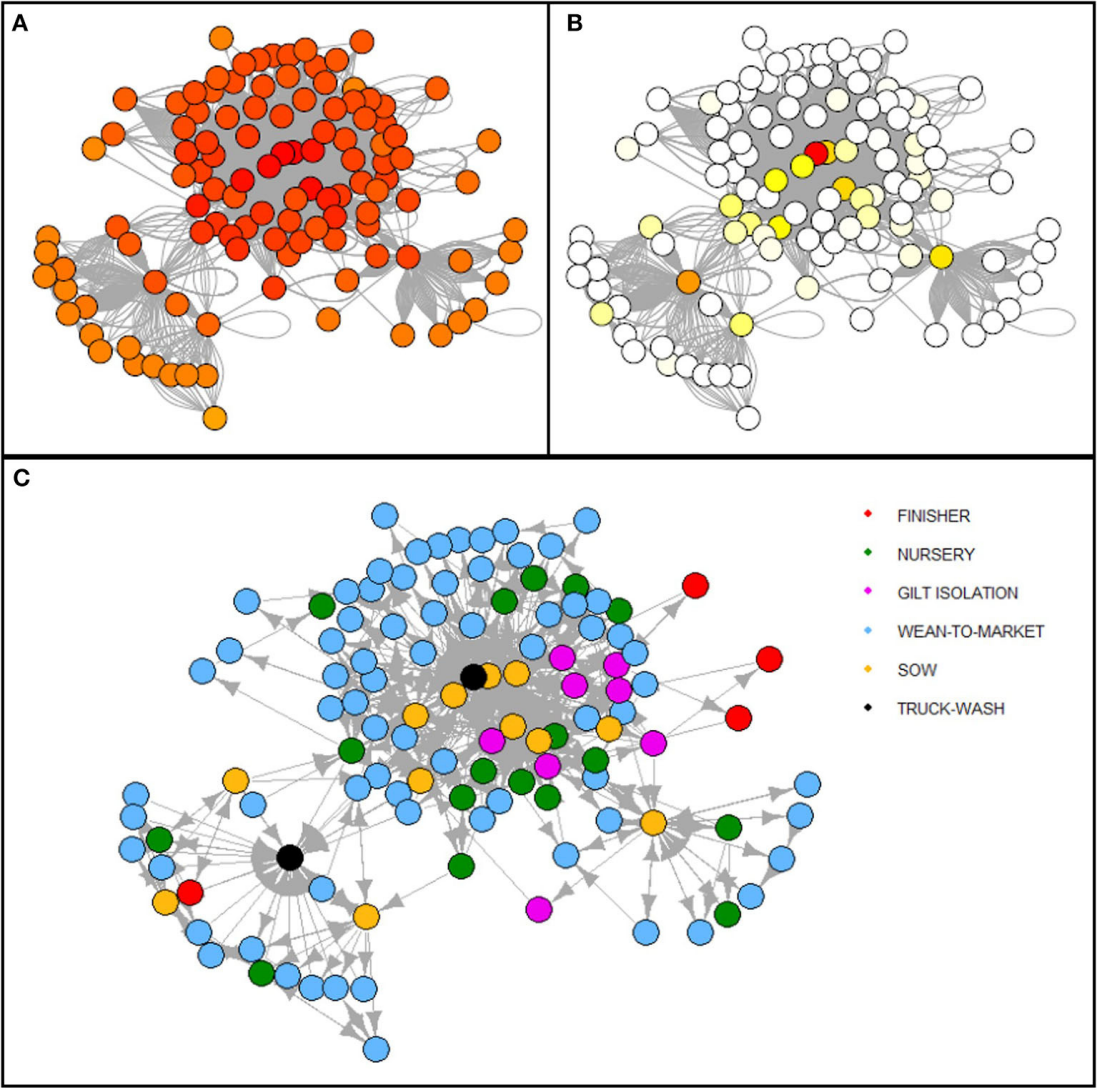
Network representation of the complete period analyzed (2019–2020). Heat scale colors represent **(A)** closeness and **(B)** betweenness relative values for each node, with the highest (red) and lowest (white) node values for each centrality metric, respectively. The five different categories of nodes and their connections are shown in different colors as reference **(C)**.

Descriptive statistics for network parameters and centrality metrics for the complete network and the network constructed for 2019 and 2020, respectively, are shown in Table 3. For the global network, on average, every site can be reached typically in three steps (*average path distance*), with less than a one-step reduction in 2019 and an increase in 2020, respectively. Although less than approximately 35% of the potential connections that could occur really occurred (*edge density*) in the complete network when assessing it on a per-year basis of the study period, the proportion doubled in 2019 compared with 2020 (43.5 vs. 25.6%). Furthermore, *degree, in-degree*, and *out-degree* were higher for 2019 than 2020 with important higher outliers observed, corresponding to truck wash facilities and sow farms. A similar tendency was observed for centrality *closeness*. However, *betweenness* showed higher values for 2020 than 2019, and the nodes with the highest values were the two truck wash facilities (Figure 4B, red and orange circles).

**Table 3:** Network parameters and centrality metrics at the node level calculated for the complete vehicle network and the 2019 and 2020 networks, respectively.

**Global parameters**	**Complete network**	**2019**	**2020**
Nodes	114	80	85
Edges	4,579	2,749	1,830
Edge density	0.3493	0.435	0.2563
Diameter	7	7	7
Average path length	3.0925	2.596	3.4837
Transitivity (global)	0.2218	0.205	0.2573
**Node parameters**	**Mean (min–Max)**	**Mean (min–max)**	**Mean (min–max)**
Degree	79.63 (2–2,199)	68.72 (2–1,546)	43.06 (2–653)
In	39.82 (1–1,095)	34.36 (1–770)	21.53(1–325)
Out	39.82 (1–1,104)	34.36 (1–776)	21.53 (1–325)
Closeness	0.0024 (7.63e-05–0.0034)	0.004 (1.6e-04–0.0058)	0.003 (1.4e-04–0.004)
In	0.0022 (7.628e-05–0.0033)	0.0036 (1.5e-04–0.0052)	0.0027 (1.4e-04–0.004)
Out	0.0022 (7.68e-05–0.0028)	0.0035 (1.5e-04–0.0048)	0.0027 (1.4e-04–0.0036)
Betweenness	234.40 (0–5694.07)	122.94 (0–3539.24)	203.72 (0–3017.81)

Complete network cluster analysis recognized three communities, and 85.3% (2,808/3,292) of the cliques observed were fully connected in pairs (i.e., cliques' size of two). When yearly analyses were performed, larger cliques with up to seven nodes fully interconnected were identified each year (Table 4). Furthermore, two and four communities were observed in 2019 and 2020, respectively, after excluding those with two or fewer members.

**Table 4:** Cliques and communities identified by the year of study (2019 and 2020).

**2019**				**2020**			
**Cliques (*****N*** = **805)**		**Community**	**Nodes member (*****N*** = **80)**	**Cliques (*****N*** = **594)**		**Community**	**Nodes member (*****N*** = **85)**
**Size**	***n***		***n*** **(n/N)**	**Size**	***n***		***n*** **(n/N)**
2	250	1	66 (0.825)	2	220	1	36 (0.424)
3	297	2	10 (0.125)	3	205	2	10 (0.118)
4	178			4	109	3	12 (0.141)
5	62			5	44	4	24 (0.282)
6	16			6	14		
7	2			7	2		

Communities with one or two members (i.e., nodes) are not included in this table.

## Discussion

This study, to the best of our knowledge, was the first description of vehicle movements using GPS data of a typical pig production system in the Midwestern US. Moreover, we have provided the first comparison between networks from a typical year of production (2019), and 2020, a year in which the swine industry was strongly affected due to the temporary decline in slaughter rates as a result of the COVID-19 pandemic in the USA (23).

A total of 74% of the records were accurate and used in these analyses. Data generated by vehicle-tracking devices have shown to be a robust tool to understand connectivity among farms, which can give a more in-depth picture of the vehicle trajectory, sites visited, and time spent at each stop. This data can serve as a proxy for compliance regarding times dedicated to animal loading/unloading, vehicle flow and vehicle cleaning, and disinfection and downtime procedures (7). The majority of the movements were performed in 2019, with a 20% reduction during the following year and strong fluctuations in the proportion of farms with weekly movements in 2020 (5–20% of the sites performing weekly movements). As expected, trucks that performed the majority of movements were those used for transporting weaned piglets that, according to production management practices, were weaned from sow farms more than a week ago. The highest reduction of vehicle movements was during spring 2020, with a follow-up increase in mid-2020, mostly due to sow and WTM movements. One potential explanation could be the limitations associated with the high rates of COVID-19 illness among workers at slaughter plants, limiting the capacity for slaughter in abattoirs during the spring of 2020 (24) and, in other cases, the temporary shutdown of major slaughter plants in the region during April and May 2020 (23). Although, movements to slaughter were not included in this study, limitations on pig-slaughter is expected to affect the pig flow within the systems in the initial levels of production, which will recover later in that year. Another potential explanation could be an increase in sites covered by the trucks in response to the initial movement reduction, despite only 5 extra nodes being observed in 2020 compared to 2019 (85/80).

As expected, a level of “fidelity” was observed within sites, with vehicles connecting sow, WTM, and truck wash facilities in the direction indicated by the production system. We observed that approximately 20% of the movements were performed from truck wash facilities to sow farms and from sow farms to WTM farms. However, approximately 14.5% of movements were connecting WTM to truck wash instead of the approximately 20% expected, which seemed to be balanced with movements from WTM to other sites, such as sow farms and WTM. Although WTM to WTM movements are common when pigs need to be finished in different sites, the movements of WTM to sow farms at the commercial level are rare as their terminal genetic makeup of the commercial market pig populations is not suitable if these animals were to be used as replacement breeding stock. Additionally, commercial growing pigs housed in WTM facilities are usually located in highly dense regions where the prevalence of endemic pathogens is high, which will represent a risk to the sow farm if these were to be introduced. WTM to sow farm movement exists but these are related to moving high-health gilts from the genetic multiplication herds that tend to have better health than commercial sow farms. Overall time spent in truck wash facilities (~3 h) denotes a sufficient time for the washing and drying process recommended for animal transport within the industry (25), with a noticeable median increase among seasons in 2020, although maximum times remain constant. This can indicate changes in disinfection procedures due to COVID-19 awareness which resulted in longer times at the truck wash facilities. It is important to mention that trucks could spend more time at the truck wash after being cleaned as they would be parked until needed again. This could bias the parameter, overestimating the mean time spent at the truck wash facilities overall. This metric needs to be further studied and analyzed but is the first insight as a proxy for biosecurity compliance. Furthermore, there were more paths connecting truck washes in 2020, which together with the increase in the median time at truck wash observed can indicate that these changes in cleaning and disinfection procedures could potentially be a result of an increase in biosecurity concerns as a whole and affected the connectivity among the network that year. The observed larger number of communities in 2020 could be reflecting management practices applied to segregate the network and avoid disease transmission between sites. While we lack data to identify the reason for observing these nodes largely interconnected (i.e., clusters, cliques, and communities), it exposes the potential usefulness of these datasets when applying control or surveillance strategies targeting groups of sites at high/low risk of infection through vehicle movements (16, 26).

Network metrics displayed a high interconnection among sites through vehicles, in which regardless of the geographical distance among farms, they were only three steps away from each other (i.e., *mean distance*). Areas with a high density of vehicle movements were detected. This finding shows that movements are not randomly distributed among all sites integrating the system, highlighting potential target areas for disease active surveillance when resources are limited as described in other production systems globally (5, 27, 28). The validity of this finding in other states is yet to be evaluated. Comparison of these network metrics with others can be challenging since systems, data collection, movements, inclusion criteria, and time frame can vary across studies (5, 29, 30). However, the need to improve within-system movement records has been reported, as it represents an important factor in disease spread (6), and our study provides information on one of the denser swine production regions within the USA, and insights are representative of the Midwestern USA swine industry.

A limitation of this study is that the network characterization was performed in one production system representative of current pig production practices in the US and might not fully represent patterns of different states or regions of the USA. Another limitation is related to the number of trucks and trailers monitored, which perhaps underestimated connectivity trends. Although a low (9.3%) proportion of company vehicles were enrolled in the study, these vehicles were those used the most and continuously during 2019–2020, resulting in a robust representation of typical movement patterns before and during the COVID-19 pandemic. In addition, the network built in this study was composed of breeding farms, growing pig farms, and truck wash facilities which leave out the connectivity with slaughter plants and market-hog transport cleaning and disinfection facilities. Furthermore, the unusual aspects observed during the COVID-19 global pandemic highlight a unique situation within the swine industry. However, this analysis builds on the understanding of the complex network interactions of vehicles within the Midwest swine industry and gives insights into how unique events can affect the flow of the pork supply production, informing preparedness for future infectious disease epidemics and events.

## Conclusion

In this study of vehicle movements within a pig production system, we observed a highly connected and structured organized network with high fidelity among the site triad of sow, WTM, and truck wash. We also described the impact on movements and network connectivity in 2020, when the peak of the COVID-19 epidemic was observed within the USA. The directionality of movements among sites and time spent at truck wash facilities represented a proxy for good biosecurity practices within the system. Monitoring and understanding spatiotemporal and network patterns of movements beforehand can prove useful in identifying high-risk areas and preparedness in the face of a new disease epidemic. The results also showed the grouping of farms within the system when considering the vehicles, indicating that many farms were connected by these indirect means. Knowledge obtained from this movement characterization in the Midwest may assist with targeting interventions, prevention, and control strategies for infectious diseases within the swine industry, with an emphasis on monitoring and reducing the prevalence in different communities connected by vehicles.

## Data availability statement

The datasets presented in this article are not readily because available are confidential. Requests to access the datasets should be directed to corzo@umn.edu.

## Author contributions

CP-R, CV, and CC: conceptualization. CV, JS, and CC: fieldwork and data collection. CC and MS: resources. CP-R: methodology and data analysis and writing the original draft. CP-R, CC, MK, CV, and JS: writing—reviewing and editing. CC: project administration. CC and CV: funding acquisition. All authors have read and agreed to the published version of the manuscript.

## References

[1] Neumann E Hall W Dahl J Hamilton D Kurian A. Is transportation a risk factor for African swine fever transmission in Australia: a review. Aust Vet J. (2021) 99:459–68. 10.1111/avj.13106 34235721

[2] Smith RP Cook AJC Christley RM. Descriptive and social network analysis of pig transport data recorded by quality assured pig farms in the UK. Prev Vet Med. (2013) 108:167–77. 10.1016/j.prevetmed.2012.08.011 22959427

[3] VanderWaal K Perez A Torremorrell M Morrison RM Craft M. Role of animal movement and indirect contact among farms in transmission of porcine epidemic diarrhea virus. Epidemics. (2018) 24:67–75. 10.1016/j.epidem.2018.04.001 29673815 PMC7104984

[4] Galvis JA Corzo CA Machado G. Modelling and assessing additional transmission routes for porcine reproductive and respiratory syndrome virus: vehicle movements and feed ingredients. Transbound Emerg Dis. (2022) 69:e1549–60. 10.1111/tbed.14488 35188711 PMC9790477

[5] Lee K Polson D Lowe E Main R Holtkamp D Martínez-López B. Unraveling the contact patterns and network structure of pig shipments in the United States and its association with porcine reproductive and respiratory syndrome virus (PRRSV) outbreaks. Prev Vet Med. (2017) 138:113–23. 10.1016/j.prevetmed.2017.02.001 28237226

[6] Machado G Vilalta C Recamonde-Mendoza M Corzo C Torremorell M Perez A. Identifying outbreaks of Porcine Epidemic Diarrhea virus through animal movements and spatial neighborhoods. Sci Rep. (2019) 9:457. 10.1038/s41598-018-36934-8 30679594 PMC6345879

[7] Dee SA Deen J Otake S Pijoan C. An experimental model to evaluate the role of transport vehicles as a source of transmission of porcine reproductive and respiratory syndrome virus to susceptible pigs. Can J Vet Res. (2004) 68:128–33. 15188957 PMC1142156

[8] Thakur KK Revie CW Hurnik D Sanchez J. Modelling contamination of trucks used in the shipment of pigs infected with porcine reproductive and respiratory syndrome virus. J Swine Health Product. (2017) 25:183–93.

[9] Alarcón LV Allepuz A Mateu E. Biosecurity in pig farms: a review. Porcine Health Manage. (2021) 7:5. 10.1186/s40813-020-00181-z 33397483 PMC7780598

[10] Chaters GL Johnson PCD Cleaveland S Crispell J de Glanville WA Doherty T. Analysing livestock network data for infectious disease control: an argument for routine data collection in emerging economies. Philos Trans R Soc Lond Ser B Biol Sci. (2019) 374:20180264. 10.1098/rstb.2018.0264 31104601 PMC6558568

[11] Lentz HHK Koher A Hövel P Gethmann J Sauter-Louis C Selhorst T. Disease spread through animal movements: a static and temporal network analysis of pig trade in Germany. PLoS ONE. (2016) 11:e0155196. 10.1371/journal.pone.0155196 27152712 PMC4859575

[12] Marquetoux N Stevenson MA Wilson P Ridler A Heuer C. Using social network analysis to inform disease control interventions. Prev Vet Med. (2016) 126:94–104. 10.1016/j.prevetmed.2016.01.022 26883965

[13] Silk MJ Croft DP Delahay RJ Hodgson DJ Boots M Weber N. Using social network measures in wildlife disease ecology, epidemiology, and management. Bioscience. (2017) 67:245–57. 10.1093/biosci/biw175 28596616 PMC5384163

[14] VanderWaal K Picasso C Enns EA Craft ME Alvarez J. Network analysis of cattle movements in Uruguay: quantifying heterogeneity for risk-based disease surveillance and control. Prev Vet Med. (2016) 123:12–22. 10.1016/j.prevetmed.2015.12.003 26708252

[15] Melmer DJ O'Sullivan TL Greer AL Poljak Z. An investigation of transportation practices in an Ontario swine system using descriptive network analysis. PLoS ONE. (2020) 15:e0226813. 10.1371/journal.pone.0226813 31923199 PMC6953787

[16] Sterchi M Faverjon C Sarasua C Vargas ME Berezowski J Bernstein A. The pig transport network in Switzerland: structure, patterns, and implications for the transmission of infectious diseases between animal holdings. PLoS ONE. (2019) 14:e0217974. 10.1371/journal.pone.0217974 31150524 PMC6544307

[17] Perez AM Linhares DCL Arruda AG VanderWaal K Machado G Vilalta C. Individual or common good? Voluntary data sharing to inform disease surveillance systems in food animals. Front Vet Sci. (2019) 6:194. 10.3389/fvets.2019.00194 31294036 PMC6598744

[18] Byers AL Allore H Gill TM Peduzzi PN. Application of negative binomial modeling for discrete outcomes: a case study in aging research. J Clin Epidemiol. (2003) 56:559–64. 10.1016/S0895-4356(03)00028-3 12873651

[19] Martínez-López B Perez AM Sánchez-Vizcaíno JM. Social network analysis. Review of general concepts and use in preventive veterinary medicine. Transbound Emerg Dis. (2009) 56:109–20. 10.1111/j.1865-1682.2009.01073.x 19341388

[20] Raghavan UN Albert R Kumara S. Near linear time algorithm to detect community structures in large-scale networks. Phys Rev E. (2007) 76:036106. 10.1103/PhysRevE.76.036106 17930305

[21] R Core Team. R: A Language Environment For Statistical Computing. Vienna: R Foundation for Statistical Computing (2017). Available online at: https://www.R-project.org/

[22] Csárdi G Nepusz T. The igraph software package for complex network research. InterJ Complex Syst. (2006) 1695.

[23] Padilla S Schulz LL Vaiknoras K MacLachlan M. COVID-19 Affected Hog Slaughter Volumes in 2020, but Impacts Trajectory of Recovery Differed Across Regions. USDA Economic Research Service (2021). Available online at: https://www.ers.usda.gov/amber-waves/2022/february/covid-19-affected-hog-slaughter-volumes-in-2020-but-impacts-and-trajectory-of-recovery-differed-across-regions/

[24] Hayes DJ Schulz LL Hart CE Jacobs KL. A descriptive analysis of the COVID-19 impacts on U.S. pork, turkey, and egg markets. Agribusiness. (2021) 37:122–41. 10.1002/agr.21674 33362337 PMC7753661

[25] Connor J Lower A Lowe J. Truck Wash Biosecurity Critical. National Hog Farmer (2014). Available online at: https://www.nationalhogfarmer.com/news/isu-economist-lee-schulz-receives-applied-research-award

[26] Dorjee S Revie CW Poljak Z McNab WB Sanchez J. Network analysis of swine shipments in Ontario, Canada, to support disease spread modelling and risk-based disease management. Prev Vet Med. (2013) 112:118–27. 10.1016/j.prevetmed.2013.06.008 23896577

[27] Martin V Zhou X Marshall E Jia B Fusheng G FrancoDixon MA. Risk-based surveillance for avian influenza control along poultry market chains in South China: the value of social network analysis. Prev Vet Med. (2011) 196–205. 10.1016/j.prevetmed.2011.07.007 21925753 PMC7127115

[28] VanderWaal K Enns EA Picasso C Alvarez J Perez A Fernandez F. Optimal surveillance strategies for bovine tuberculosis in a low-prevalence country. Sci Rep. (2017) 7:4140. 10.1038/s41598-017-04466-2 28646151 PMC5482878

[29] Arruda AG Friendship R Carpenter J Hand K Poljak Z. Network, cluster and risk factor analyses for porcine reproductive and respiratory syndrome using data from swine sites participating in a disease control program. Prev Vet Med. (2016) 128:41–50. 10.1016/j.prevetmed.2016.03.010 27237389

[30] Büttner K Krieter J Traulsen A Traulsen I. Static network analysis of a pork supply chain in Northern Germany-Characterisation of the potential spread of infectious diseases via animal movements. Prev Vet Med. (2013) 110:418–28. 10.1016/j.prevetmed.2013.01.008 23462679

