# Supplementary material for: Disentangling transport movement patterns of trucks either transporting pigs or while empty within a swine production system before and during the COVID-19 epidemic
Source: Front Vet Sci. 2023 Jul 14;10:1201644. doi: 10.3389/fvets.2023.1201644 (PMC10376687; doi:10.3389/fvets.2023.1201644)
Supplement: Supplementary file 1 [file Table_1.docx]

**Appendix A: Supplementary material**

**Supplementary Table S1:** Results of multivariate negative binomial regression, with adjustment for multiple comparisons comparing frequency of in-movements by year, season, and farm production type.

| Season-Yr | Production_type | Ratio | SE | 95% LCI | 95% UCI | p.value |
| --- | --- | --- | --- | --- | --- | --- |
| Fall19 | FINISHER | 2.688 | 0.212 | 2.159 | 3.347 | <.0001 |
| Fall19 | GILT ISOLATION | 50.502 | 17.757 | 18.999 | 134.239 | <.0001 |
| Fall19 | NURSERY | 95.448 | 33.181 | 36.307 | 250.923 | <.0001 |
| Fall19 | WEAN-TO-MARKET | 326.568 | 112.502 | 125.311 | 851.060 | <.0001 |
| Fall19 | TRUCK WASH | 372.604 | 128.304 | 143.036 | 970.621 | <.0001 |
| Fall19 | SOW | 521.033 | 179.260 | 200.181 | 1356.151 | <.0001 |
| Fall20 | FINISHER | 2.281 | 0.184 | 1.824 | 2.854 | <.0001 |
| Fall20 | GILT ISOLATION | 42.857 | 15.085 | 16.107 | 114.036 | <.0001 |
| Fall20 | NURSERY | 80.998 | 28.188 | 30.779 | 213.159 | <.0001 |
| Fall20 | WEAN-TO-MARKET | 277.131 | 95.571 | 106.234 | 722.951 | <.0001 |
| Fall20 | TRUCK WASH | 316.198 | 108.995 | 121.262 | 824.509 | <.0001 |
| Fall20 | SOW | 442.157 | 152.279 | 169.710 | 1151.980 | <.0001 |
| Spring19 | FINISHER | 2.662 | 0.210 | 2.137 | 3.316 | <.0001 |
| Spring19 | GILT ISOLATION | 50.012 | 17.586 | 18.814 | 132.944 | <.0001 |
| Spring19 | NURSERY | 94.521 | 32.861 | 35.953 | 248.502 | <.0001 |
| Spring19 | WEAN-TO-MARKET | 323.399 | 111.417 | 124.088 | 842.848 | <.0001 |
| Spring19 | TRUCK WASH | 368.989 | 127.066 | 141.641 | 961.255 | <.0001 |
| Spring19 | SOW | 515.977 | 177.530 | 198.227 | 1343.062 | <.0001 |
| Spring20 | GILT ISOLATION | 18.787 | 6.437 | 7.247 | 48.706 | <.0001 |
| Spring20 | NURSERY | 35.507 | 12.020 | 13.852 | 91.013 | <.0001 |
| Spring20 | WEAN-TO-MARKET | 121.484 | 40.728 | 47.830 | 308.561 | <.0001 |
| Spring20 | TRUCK WASH | 138.610 | 46.446 | 54.598 | 351.894 | <.0001 |
| Spring20 | SOW | 193.826 | 64.881 | 76.420 | 491.605 | <.0001 |
| Summer19 | FINISHER | 2.902 | 0.227 | 2.335 | 3.607 | <.0001 |
| Summer19 | GILT ISOLATION | 54.525 | 19.163 | 20.522 | 144.871 | <.0001 |
| Summer19 | NURSERY | 103.051 | 35.808 | 39.216 | 270.796 | <.0001 |
| Summer19 | WEAN-TO-MARKET | 352.583 | 121.412 | 135.348 | 918.480 | <.0001 |
| Summer19 | TRUCK WASH | 402.286 | 138.466 | 154.493 | 1047.516 | <.0001 |
| Summer19 | SOW | 562.538 | 193.460 | 216.213 | 1463.602 | <.0001 |
| Summer20 | FINISHER | 2.017 | 0.165 | 1.606 | 2.533 | <.0001 |
| Summer20 | GILT ISOLATION | 37.894 | 13.350 | 14.228 | 100.920 | <.0001 |
| Summer20 | NURSERY | 71.618 | 24.947 | 27.190 | 188.643 | <.0001 |
| Summer20 | WEAN-TO-MARKET | 245.037 | 84.581 | 93.848 | 639.790 | <.0001 |
| Summer20 | TRUCK WASH | 279.580 | 96.460 | 107.124 | 729.663 | <.0001 |
| Summer20 | SOW | 390.951 | 134.765 | 149.927 | 1019.451 | <.0001 |
| Winter19 | FINISHER | 2.515 | 0.200 | 2.016 | 3.138 | <.0001 |
| Winter19 | GILT ISOLATION | 47.254 | 16.622 | 17.770 | 125.656 | <.0001 |
| Winter19 | NURSERY | 89.309 | 31.060 | 33.958 | 234.879 | <.0001 |
| Winter19 | WEAN-TO-MARKET | 305.566 | 105.309 | 117.206 | 796.633 | <.0001 |
| Winter19 | TRUCK WASH | 348.641 | 120.101 | 133.786 | 908.545 | <.0001 |
| Winter19 | SOW | 487.524 | 167.797 | 187.236 | 1269.408 | <.0001 |
| Winter20 | FINISHER | 1.961 | 0.161 | 1.560 | 2.466 | <.0001 |
| Winter20 | GILT ISOLATION | 36.849 | 12.985 | 13.833 | 98.158 | <.0001 |
| Winter20 | NURSERY | 69.643 | 24.265 | 26.434 | 183.481 | <.0001 |
| Winter20 | WEAN-TO-MARKET | 238.280 | 82.267 | 91.240 | 622.283 | <.0001 |
| Winter20 | TRUCK WASH | 271.870 | 93.822 | 104.148 | 709.696 | <.0001 |
| Winter20 | SOW | 380.170 | 131.078 | 145.761 | 991.551 | <.0001 |
| Winter21 | FINISHER | 0.249 | 0.036 | 0.167 | 0.372 | <.0001 |
| Winter21 | GILT ISOLATION | 4.687 | 1.741 | 1.668 | 13.168 | 0.0003 |
| Winter21 | NURSERY | 8.858 | 3.258 | 3.186 | 24.628 | <.0001 |
| Winter21 | WEAN-TO-MARKET | 30.307 | 11.054 | 10.993 | 83.552 | <.0001 |
| Winter21 | TRUCK WASH | 34.579 | 12.607 | 12.548 | 95.288 | <.0001 |
| Winter21 | SOW | 48.354 | 17.612 | 17.563 | 133.123 | <.0001 |

Contrast variable: Spring 2020, and finisher production type.

Winter21 refers to December 2020.

**Supplementary Table S2:** Frequency of in-movements by farm-type (in percentage) and dyads. Graded scale colors represent a higher (red) and lower (white) percentage of movements across dyad connections by year and season based on 2749 and 1830 movements in 2019 and 2020 respectively.

|  |  | **2019** | | | | | | **2020** | | | | | |
| --- | --- | --- | --- | --- | --- | --- | --- | --- | --- | --- | --- | --- | --- |
| **Origin\|Destination** | | **GDU** | **NURSERY** | **SOW** | **TRUCK WASH** | **WTM** | **FINISHER** | **GDU** | **NURSERY** | **SOW** | **TRUCK WASH** | **WTM** | **FINISHER** |
| **SPRING** | **FINISHER** | 0.04 | 0.00 | 0.00 | 0.00 | 0.00 | 0.00 | 0.00 | 0.00 | 0.00 | 0.00 | 0.00 | 0.00 |
|  | **GDU** | 0.33 | 0.00 | 0.51 | 0.04 | 0.00 | 0.04 | 0.11 | 0.05 | 0.55 | 0.00 | 0.00 | 0.00 |
|  | **NURSERY** | 0.00 | 0.29 | 0.07 | 0.73 | 0.07 | 0.00 | 0.11 | 0.00 | 0.11 | 0.44 | 0.05 | 0.00 |
|  | **SOW** | 0.44 | 0.77 | 0.80 | 1.35 | 5.48 | 0.00 | 0.33 | 0.38 | 1.59 | 1.81 | 1.92 | 0.00 |
|  | **TRUCK WASH** | 0.11 | 0.04 | 5.81 | 0.84 | 0.33 | 0.00 | 0.16 | 0.16 | 2.75 | 0.55 | 0.38 | 0.00 |
|  | **WTM** | 0.00 | 0.07 | 1.79 | 3.98 | 0.18 | 0.00 | 0.00 | 0.05 | 1.15 | 1.21 | 0.00 | 0.00 |
| **SUMMER** | **GDU** | 0.22 | 0.04 | 0.29 | 0.00 | 0.00 | 0.00 | 0.22 | 0.05 | 0.44 | 0.05 | 0.05 | 0.00 |
|  | **NURSERY** | 0.04 | 0.58 | 0.33 | 1.24 | 0.04 | 0.00 | 0.11 | 0.11 | 0.22 | 0.77 | 0.16 | 0.00 |
|  | **SOW** | 0.22 | 1.35 | 0.91 | 1.57 | 5.44 | 0.00 | 0.27 | 0.93 | 3.29 | 2.09 | 5.33 | 0.00 |
|  | **TRUCK WASH** | 0.07 | 0.15 | 6.21 | 0.66 | 0.51 | 0.00 | 0.22 | 0.16 | 5.11 | 0.33 | 0.66 | 0.00 |
|  | **WTM** | 0.00 | 0.15 | 1.90 | 4.09 | 0.26 | 0.00 | 0.00 | 0.05 | 2.86 | 3.35 | 0.33 | 0.00 |
| **FALL** | **FINISHER** | 0.04 | 0.00 | 0.00 | 0.00 | 0.00 | 0.00 | 0.05 | 0.05 | 0.11 | 0.16 | 0.00 | 0.00 |
|  | **GDU** | 0.66 | 0.00 | 0.55 | 0.00 | 0.00 | 0.04 | 0.27 | 0.00 | 0.49 | 0.00 | 0.00 | 0.11 |
|  | **NURSERY** | 0.07 | 0.51 | 0.26 | 0.66 | 0.04 | 0.00 | 0.05 | 0.27 | 0.82 | 0.77 | 0.22 | 0.00 |
|  | **SOW** | 0.37 | 0.80 | 0.95 | 1.35 | 5.48 | 0.00 | 0.33 | 1.32 | 2.20 | 2.03 | 6.53 | 0.27 |
|  | **TRUCK WASH** | 0.11 | 0.26 | 5.70 | 0.47 | 0.29 | 0.00 | 0.16 | 0.11 | 5.60 | 0.88 | 0.88 | 0.00 |
|  | **WTM** | 0.00 | 0.00 | 1.46 | 4.35 | 0.29 | 0.00 | 0.00 | 0.44 | 3.57 | 3.62 | 0.27 | 0.00 |
| **WINTER** | **GDU** | 0.58 | 0.00 | 0.33 | 0.00 | 0.00 | 0.00 | 0.77 | 0.00 | 0.55 | 0.05 | 0.00 | 0.00 |
|  | **NURSERY** | 0.00 | 0.80 | 0.29 | 1.32 | 0.04 | 0.00 | 0.05 | 0.38 | 0.38 | 0.88 | 0.00 | 0.00 |
|  | **SOW** | 0.26 | 1.21 | 0.88 | 0.77 | 4.75 | 0.00 | 0.33 | 1.26 | 1.37 | 1.26 | 4.12 | 0.00 |
|  | **TRUCK WASH** | 0.04 | 0.29 | 5.22 | 0.40 | 0.33 | 0.00 | 0.22 | 0.05 | 4.67 | 0.77 | 1.10 | 0.00 |
|  | **WTM** | 0.04 | 0.11 | 1.10 | 3.84 | 0.37 | 0.00 | 0.00 | 0.05 | 1.37 | 3.79 | 0.38 | 0.00 |
